# Quality of the delivery services in health facilities in Northern Ethiopia

**DOI:** 10.1186/s12913-017-2125-3

**Published:** 2017-03-09

**Authors:** Girmatsion Fisseha, Yemane Berhane, Alemayehu Worku, Wondwossen Terefe

**Affiliations:** 1 School of Public Health, Mekelle University, Mekelle, Ethiopia; 2 Addis Continental Institute of Public Health, Addis Ababa, Ethiopia; 3 School of Public Health, Addis Ababa University, Addis Ababa, Ethiopia

**Keywords:** Quality, Delivery, Newborn, Care, Northern Ethiopia

## Abstract

**Background:**

Substantial improvements have been observed in the coverage of and access to maternal health service, especially in skilled birth attendants, in Ethiopia. However, the quality of care has been lagging behind. Therefore, this study investigated the status of the quality of delivery services in Northern Ethiopia.

**Methods:**

A facility based survey was conducted from December 2014 to February 2015 in Northern Ethiopia. The quality of delivery service was assessed in 32 health facilities using a facility audit checklist, by reviewing delivery, by conducting in-depth interview and observation, and by conducting exit interviews with eligible mothers. Facilities were considered as ‘good quality’ if they scored positively on 75% of the quality indicators set in the national guidelines for all the three components; input (materials, infrastructure, and human resource), process (adherence to standard care procedures during intrapartum and immediate postpartum periods) and output (the mothers’ satisfaction and utilization of lifesaving procedures).

**Results:**

Overall 2 of 32 (6.3%) of the study facilities fulfilled all the three quality components; input, process and output. Two of the three components were assessed as good in 11 of the 32 (34.4%) health facilities. The input quality was the better of the other quality components; which was good in 21 out of the 32 (65.6%) health facilities. The process and output quality was good in only 10 of the 32 (31.3%) facilities.

**Conclusions:**

Only 6.3% of the studied health facilities had good quality in all three dimensions of quality measures that was done in accordance to the national delivery service guidelines. The most compromised quality component was the process. Systematic and sustained efforts need to be strengthened to improve all dimensions of quality in order to achieve the desired quality of delivery services and increase the proportion of births occurring in health facilities.

**Electronic supplementary material:**

The online version of this article (doi:10.1186/s12913-017-2125-3) contains supplementary material, which is available to authorized users.

## Background

In low- and middle-income countries, including Ethiopia, childbirth poses significant risks to mothers and newborns [1]. Poor quality of care contributes significantly to a high maternal and newborn mortality rate at the health facility level [2]. In Ethiopia, 35.5% of maternal deaths that occurs in hospitals are related to medical errors and inadequate hospital service such as lack of blood for transfusion, delay in transfusion, and inappropriate treatment [3–5]. Although infrastructure expansion was phenomenal in Ethiopia in the last decade, some heath facilities were made operational without the necessary materials and the necessary human resources, which cast doubts about the quality of services rendered in the rapidly expanding facilities. Health facilities can only provide quality services if their physical infrastructure is matched with adequate and functional materials and supplies, if they sufficient number of trained human resources that are performing up to standards, and if their target population is satisfied with the services and continue utilizing the services.

In Ethiopia about 15% of births were attended by skilled birth attendants (SBAs) nationally, though state level variations were very wide [6]. SBA utilization in northern Ethiopia, where this study was conducted, has increased from 6% in 2005 to 24.7% in 2014 [6, 7]. Improving quality of obstetric care and increasing the number of deliveries attended by skilled personnel to 80% by 2020 is one of the strategies of the health sector plan of Ethiopia [8].

The reasons for not delivering in health facilities in Ethiopia include perceived and actual poor quality of services, facility not regularly open for services, and unavailability of female providers [7, 9]. Ethiopia has developed and implemented a comprehensive national guideline to ensure the provision of quality institutional delivery services at all levels of the health facilities. The guidelines clearly indicate the Donabedian model three quality components that considers input (materials, infrastructure and human resources), process (adherence to standard care during intrapartum and immediate postpartum periods) and output (satisfaction of mothers and utilization of emergency obstetric and newborn care (EmONC)). However, very few studies tried to assess the quality of delivery services using all the three components of the Donabedian model [10–14]. Therefore, this study used the Donabedian model to assess the overall quality of delivery services in health facilities in northern Ethiopia.

## Methods

The study was conducted in three zones of Tigray region (East, South and South East) in Northern Ethiopia involving 21 districts. A total of 90 health centers and 5 hospitals were present in the zone at the time of the study. Approximately 2.5 million people were expected to live in the catchment area. The Total Fertility Rate in the region is 4.6% and the institutional delivery rate is 11.6% [9]. Delivery service in the region is provided at the hospital, mostly led by an obstetrician, and in health centers mostly led by midwives, emergency obstetric surgeons, health officers or nurses. The service is given free of charge in all public health facilities and the service is supposed to be open 24 h.

This study was a facility based cross-sectional study that was conducted between December 2014 and February 2015. The study participants included health care providers and mothers. Providers including the health facility heads, and skilled birth attendants (midwives and nurses) were interviewed. Mothers identified in the active first stage of labour during the study period were observed until discharged and interviewed at exit.

A total of 32 health facilities (27 health centers and all the five hospitals) were selected for the study. Health facilities were selected randomly, instead of using criteria based selection procedure, in each study zone proportional to the total number of health facilities existing in each zone since the type of infrastructure and the human resource deployment procedures were similar in the study areas. All skilled birth attendants in the selected facilities were included in the study. Mothers attending the delivery service were enrolled consecutively during the study period for observation, exit interview and chart review.

Five types of data collection tools were used to gather data for this study. Facility audit form was used to interview the head of the facilities about availability of essential equipment, drugs and supplies at the time of the survey. A mother’s exit interview questionnaire was used to gather information about the process of delivery and perceived quality of services and their satisfaction. A record review form was used to gather data from the maternity registers about the utilization of emergency obstetric and newborn care (EmONC) during the three months prior to the study period see Additional file 1 and 2. A semi-structured interview guide was used to conduct in-depth interviews with skilled birth attendants about their experiences. Non-participatory observation was made using a checklist to observe mothers and SBAs during childbirth and immediate postpartum periods to assess adherence to the standard practice.

Data were collected by midwives and nurses who had more than two years work experience in maternal health service and three supervisors with a master’s degree. Training was provided for data collectors and supervisors. During the training, the data collectors were standardized by asking them to complete the questionnaires based on mock interviews. During the data collection period there was a strict supervision scheme. Completed questionnaires were checked on a daily basis.

### Input quality

Input quality was measured using a total of 40 items which was developed from national guidelines [15, 16]. Items had good internal consistency (α = 0.688). We gave equal weight to all assessed items. Facilities were categorized as good quality if scored 75% or more of the input quality score. See the list of variables used in measuring input quality (Additional file 1).

### Process quality

Process quality was measured using 92 items which were taken from the WHO guidelines which is similar to the national guideline [17, 18]. Items included activities during examination of mothers at admission, care during the various stages of labour (first, second, third) and immediate postpartum period, interpersonal relationship, and universal hygiene precautions. Items had strong internal consistency (α = 0.925). All the items had equal weight. The individual mothers’ score was linked to the facility level by calculating the average score the mothers cared at the facility. Then, facilities were categorized as having good process quality if they score 75% or more of process criteria. See the list of variables used in measuring process quality (Additional file 1).

### Output quality

Output quality was assessed by considering both satisfaction of mother’s and EmONC utilization at the facility level. A total of 26 Likert scale items that ranged from disagree (1) to agree (3) were adapted from the national guidelines to assess satisfaction levels of mothers [10, 19]. The scales had strong internal consistency (α = 0.84). Items were given equal weight. The standard care at the mothers’ level was linked to the facility level by calculating the average score for each facility. Then, facilities were categorized as providing satisfied service to mothers if they score at least 75% of the satisfaction score. A checklist with nine items (signal functions) was prepared to assess utilization of EmONC during the three months preceding the study period, similar to the WHO recommendation [20]. The total score for health centers is ranging from 0 to 7 (basic EmONC) and for hospitals the total score is ranging from 0 to 9 scores (comprehensive EmONC). Facilities were categorized as utilizing EmONC if health centers fulfill at least 75% of the EmONC score. The two output quality scores were then combined to produce the overall output quality score. The facilities were categorized as a good performer if they had scored at least 75% on both output components. See the list of variables used in measuring output quality (Additional file 1).

### Overall quality of delivery service

This was assessed by combining input, process and output quality delivery service at the facility level. A facility is classified as providing standard quality of delivery care if it had scored at least 75% in all the three components, otherwise classified as performing below the standard. See list of each quality scores for each facilities (Additional file 1).

## Results

A total of 32 health facilities (27 health centers and 5 hospitals) were included in the study. All health facilities invited to the study agreed to participate. A total of 106 service providers in the selected facilities and 216 mothers visiting the study health facilities during the study period were enrolled into the study. All service providers and mothers who were invited to the study agreed to participate.

The quality was assessed in three components; input, process and output. Out of the 32 health facilities assessed, 21 (65.62%) fulfilled at least 75% of the input items specified in the national guidelines and were rated as good. On the process quality, 10 of the 32 (31.25%) health facilities that fulfilled at least 75% of the process criteria set in the national guidelines were rated as Good. On the output quality; 10 of the 32 (31.25%) health facilities that fulfilled at least 75% of the output criteria set in the national guidelines were rated as Good (Table 1).

**Table 1:** Delivery Service Quality category of health facilities in three quality dimension, Northern Ethiopia

Quality component	Good (scored >75%)	Not Good (Scored <75%)	Total
	Number of facilities (%)	Number of facilities (%)	
Input	21 (65.62%)	11 (34.38%)	32
Process	10 (31.25%)	22 (68.75%)	32
Output	10 (31.25%)	22 (68.75%)	32

Most health facilities lack critical input that are necessary to save a newborn life. Radiant warmer was available in 37.5% the facilities; towels to wrap newborns was available in 15.6% of the health facilities; and only 12.5% of the health facilities had trained service providers to perform newborn resuscitation. Critical inputs to ensure the wellbeing of delivering mothers was also missing in considerable proportion of health facilities. Functional transport facility necessary for referral function was available in 59.4% of the health facilities; trained providers that can handle obstetric emergency were available in 56.3% of the health facilities; service providers were actively prepared to handle delivery at any given time in 53.1% of the health facilities; and the number of midwives was as per the national guidelines in 50% of the health facilities (Table 2).

**Table 2:** Delivery service input quality items not fulfilled in at least 75% of the health facilities, Northern Ethiopia

Input quality items	Number of facilities	Percent of facilities
Laboratory service available and functional	23	71.9
The facility has running water	21	65.6
The facility has reliable electric power	21	65.6
Refrigerator available and functional	21	65.6
Personal protective items available	20	62.5
Emergency antibiotics available	20	62.5
Transport service available and functional	19	59.4
All service providers in the delivery room trained to manage obstetric emergency	18	56.3
Episiotomy set available	17	53.1
Service provider prepared to handle delivery at the facility any time (available at the facility, dressed and prepared material for delivery)	17	53.1
Midwifery number standard per national guideline (3 in HC and 13 in hospital)	16	50
The facility has three or more rooms for maternity related services	13	40.6
Radiant warmer available and functional	12	37.5
Functional toilet and shower available in maternity service area	11	34.4
Antiseptic solution available	5	15.6
At least two cloth/towel to dry or warp baby after birth available	5	15.6
The facility has a working phone or shortwave radio	4	12.5
All service providers in delivery room trained to do newborn resuscitation	4	12.5

Some of the key newborn interventions were not implemented in all health facilities; tetracycline eye ointment and vitamin K were offered in 46.9% of the health facilities. Some of the common limitations to the quality of maternity services include that women were greeted on arrival in 46.9% of the health facilities, women were encouraged to ask questions in 34.4% of the health facilities, and procedures to be undertaken were explained to the women in 18.8% of the facilities. Moreover, some of the vital signs and infection prevention procedures were not observed; partograph was used to check the progress of labor in 25.0% of the facilities, temperature was checked in 21.9% of the facilities, hand washing was practiced in 21.9% of the health facilities, respiratory rate was recorded in 12.5% of the facilities, and pulse rate was recorded in 9.4%of the facilities (Table 3).

**Table 3:** Delivery service process quality not fulfilled by at least 50% of the health facilities, Northern Ethiopia

Process quality items	Number of facilities	Percent of facilities
Greeted the woman and her companion (if present) in a cordial manner	15	46.9
Provided tetracycline eye ointment 1% prophylaxis to newborn	15	46.9
Administered vitamin k to newborn	15	46.9
Uterine contractions assessed how frequently is occurring first stage of labour	14	43.8
Identified degree of decent by abdominal palpation	13	40.6
Assessed molding	13	40.6
Did not rupture of membranes routinely during second stage of labour	13	40.6
Encourage women to walk around during the first stage of labour	13	40.6
Recorded the time of birth on partograph	13	40.6
Checked uterine contraction after birth	13	40.6
Service provider asks mother when the painful regular uterine contractions began	12	37.5
If membranes ruptured: asked when, what color and smell what it had	11	34.4
Encouraged the woman to ask questions	11	34.4
Asked if the women experienced vaginal bleeding, fever, severe headaches, blurred vision	10	31.3
Providers washed hands with running water and soap after conducting delivery	10	31.3
Asked the woman if she has urinated & encouraged her to do so during the admission/first stage of labour	10	31.3
Measured fundal height	8	25.0
Used partograph to follow the progress of labour	8	25.0
Cleansed the vulva with antiseptic solution before performing vaginal examination	8	25.0
Removed gloves after being immersed in 0.5% chlorine solution after conducting delivery	8	25.0
Providers washed hands with running water & soap after examining mothers during admission	7	21.9
Checked temperature during the admission/first stage of labour	7	21.9
Prior to conducting delivery, providers washes hands with running water and soap	7	21.9
Explain each procedure to the mother	6	18.8
Determine pulse rate during admission/first stage of labour	6	18.8
Gloves are removed after being immersed in 0.5% chlorine solution after performing each vaginal examination	6	18.8
Checked bladder distension	6	18.8
Mother health condition checked during immediate postpartum periods before discharge	6	18.8
Determined respiratory rate during the admission/first stage of labour	4	12.5
Determine pulse rate during immediate postpartum period before discharge	3	9.4

**Fig. 1:**
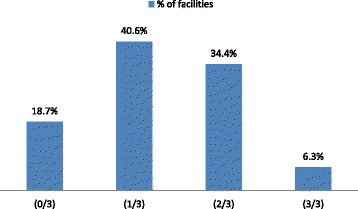
Summary of the overall delivery service quality, North Ethiopia. Note: (0/3) facilities that did not have good quality in any of the three quality components; (1/3) facilities that have good quality in only one of the three quality components; (2/3) facilities that have good quality in two of the three quality components; and (3/3) facilities that have good quality in all three quality components

The overall quality of the delivery services was determined by considering all three quality assessment components. Accordingly, only 2 of the 32 (6.25%) of the facilities were rated as offering good quality delivery services in all three quality components, while 6 of the 32 (18.75%) of the facilities were not rated as good in any of the three quality components. Only 11 of the 32 (34.37%) of the facilities were rated in at least two of the three quality components (Fig. 1).

## Discussion

We assessed the quality of delivery and newborn services in Northern Ethiopia using the three quality components suggested in the Donabedian model based on the national guidelines for the delivery service provision in Ethiopia. Accordingly, 65.62% of the surveyed health facilities were rated as good in terms of input quality, and 31.25% were good for the process and output quality. Overall, only 6.3% of the health facilities were rated as good in all the three quality components; that is input, process and output.

This study showed the overall quality of the delivery service is not in par with that described in the national guidelines. The better of the three quality components was the input. However, it is important to note that the three components are interlinked to each other and if even one component is defective the overall quality is affected [14]. In measuring each of the quality components, overestimations of findings could happen due to the either Hawthorn or social desirability biases during observations especially in measuring process quality and client satisfaction. Thus, actual quality could even be worse than reported in this paper. This study reported lower delivery quality in health facilities compared to some other African countries [12]. However, direct comparison is perhaps inappropriate due to the variations in the national guidelines and the methods used for conducting assessments. Adapting a uniform service quality guideline that is applicable in similar settings is desirable to ensure the same good quality services and also to ease measurement challenges.

Our study shows that 65.6% of the health facilities had the necessary input to provide quality delivery services. This means that essential drugs, equipment, supplies, and trained human power in adequate number were available in two-third of the health facilities. Similarly, a study done in South-Central Ethiopia reported input for quality delivery service was the better of the other two quality components for delivery service [11].

In this study only 31.25% of the facilities fulfilled the quality standards for delivery services according to the national guidelines, which comparable to reports from other Sub-Saharan Africa countries [12, 13, 21]. The poor performance of health facilities on process quality has serious implications on the efficiency of the care provided in the health facilities, leading to delayed treatment that in turn negatively affect the outcome of birth leading to either fetal/newborn or maternal death [20]. The process quality component is the least achieved quality component in this study. Unless adequate emphasis is given to improve the utilization of institutional delivery services may remain low in Ethiopia and the desired newborn and maternal health improvement may take longer time than currently aspired in the national plan [8].

Only 31.25% of the facilities were rated as good on output quality for delivery services in terms of utilization of EmONC and satisfaction of mothers. This means lifesaving skills are lacking in many health facilities and they simply rely on referring mothers to higher level health institutions. Although emergency obstetric surgeons were available in many health facilities lack of efficient blood transfusion services and anesthesiologist and poorly equipped operating theatre and unreliable supply of electricity preclude the effectiveness of the surgeons. Similar findings were reported in South Ethiopia [22] and in Ghana [12]. This indicates that input must be organized as a package rather than in pieces to effectively utilize the meager resources available in low income settings. Otherwise, idle human resources in health facilities could just be a waste of resources and a burden to the health system.

The internal consistency of each of study instruments used to assess each component of the delivery service was strong. As a limitation, in this study there may be observer bias between data collectors. Maternity staffs would have been more mindful of the presence and purpose of the observer than the laboring women. So, Hawthorne effect could be possible. Since SBAs can perform and follow the standard delivery practice for a short period of time. This condition might overestimate the process quality care. But during the long period of observation, it is difficult for the health care personnel maintain their pretended behaviors [23]. In addition, we have excluded the first few observations for each SBA during childbirth care to minimize the Hawthorne effect. In measuring satisfaction, mothers are unlikely to speak about their experiences of care as they might at their own home because it often elicits too positive response. Measuring satisfaction using only quantitative approach may not also adequate to see the feeling of mothers.

## Conclusions

Overall, 6.3% facilities met the quality criteria for essential delivery care. Only 11/32 (34.4%) facility scored at least 75% in two of the three quality components. The most compromised quality component was the process; the desired maternal and newborn services were not rendered in considerable proportion of the health facilities. Systematic and sustained efforts need to be strengthened to achieve the desired improvements in maternal and newborn health in the study context.

## Additional files

Additional file 1: Excel file showing the details scores for the three quality components. (XLS 60 kb)
Additional file 2: Copies of interview and observation guides used in this study. (PDF 217 kb)

## Abbreviations

EmONC: Emergency obstetric and newborn care
HC: Health center
SBAs: Skilled birth attendants
