# Supplementary material for: Quality of the delivery services in health facilities in Northern Ethiopia
Source: BMC Health Serv Res. 2017 Mar 9;17:187. doi: 10.1186/s12913-017-2125-3 (PMC5345168; doi:10.1186/s12913-017-2125-3)
Supplement: Additional file 2: — Copies of interview and observation guides used in this study. (PDF 217 kb) [file 12913_2017_2125_MOESM2_ESM.pdf]

Additional file 2: Showing the copies of interview and observation guides used in measuring input (Table 1), process (Table 2) and output (Table 3 and 4) quality delivery service in Northern Ethiopia study.

Table 1: Checklist used in measuring input quality delivery service in the Northern Ethiopia

**Input variables (yes/no question)**	**Response(observed)**	
Infrastructures indicators		
1. Clean water source available	1. Yes	2. No
2. Reliable electricity available	1. Yes	2. No
3. Means of communication available all times even not locked	1. Yes	2. No
4. 24 hour service available	1. Yes	2. No
5. Functioning transport facilities (Ambulance) available	1. Yes	2. No
6. At least three rooms available for maternity service	1. Yes	2. No
7. Functional refrigerator for storage of drugs and vaccines available	1. Yes	2. No
8. Mother toilet with shower service available	1. Yes	2. No
Basic equipments and supplies in the delivery room		
1. Functional Blood pressure apparatus available	1. Yes	2. No
2. Functional Oral or axillary Thermometer available	1. Yes	2. No
3. Functional Stethoscope available	1. Yes	2. No
4. Functional Fetal stethoscope available	1. Yes	2. No
5. Functional Baby weighing scales available	1. Yes	2. No
6. Sterilizers (autoclave or dry oven) available	1. Yes	2. No
7. Personal protective equipments (at least mouth mask, boots, delivery gown, apron, sterile glove) available	1. Yes	2. No
8. Antiseptics (soap, chlorine solution, alcohol, iodine)	1. Yes	2. No
9. Container for infection prevention (decontamination container, safety box, covered contaminated waste bin) available	1. Yes	2. No
10. Functional movable delivery light available	1. Yes	2. No
11. Functional Vacuum extractor available	1. Yes	2. No
Essential obstetric equipments (absolute minimum equipments for delivery)		
1. Two sterilized delivery sets (cord scissors, cord tie, two artery forceps) available	1. Yes	2. No
2. Episiotomy set (catgut, one tissue forceps, one needle holder, one scissor/blade) available	1. Yes	2. No
Consumable supplies		
1. IV set and Canula available	1. Yes	2. No
2. Folly catheter available	1. Yes	2. No
3. Blank partograph available	1. Yes	2. No
4. HIV test kit available	1. Yes	2. No
5. IV fluid (normal saline) available	1. Yes	2. No
Emergency drugs for maternal care available		
1. Oxytocin drug available	1. Yes	2. No
2. Antibiotics (at least Ampicillin, Gentamicin and Metrandazol) available	1. Yes	2. No
3. Anticonvulsant drugs (at least Magnisiaum sulfate(MgSo4)) available	1. Yes	2. No
4. Antihypertensive drug (Hydralazine) available	1. Yes	2. No
Essential Newborn care equipments and drugs		
1. Newborn resuscitation materials (at least Bag and mask, mucus extractors, mask) available	1. Yes	2. No
2. At least two towel to dry and warp baby after delivery available	1. Yes	2. No
3. Functional incubator (enough light or radiant warmer) available	1. Yes	2. No
4. Vitamin K available	1. Yes	2. No
5. Tetracycline (TTC) eye ointment available	1. Yes	2. No
Functioning Laboratory service		
1. Basic blood and Urine analysis test available (Hgb or Hct, WBC count, ABO blood group and Rh test, malaria and HIV tests, Urine analysis like protein urea,	1. Yes	2. No
Human resource for maternity service available		
2. At least three midwife available at Health Center or at least 13 midwives at hospital (adequacy of SBAs)	1. Yes	2. No
3. Skilled birth attendance(SBAs) readiness (available and properly dressed at delivery room)	1. Yes	2. No
4. All SBAs in the facility trained to manage obstetric complication	1. Yes	2. No
5. At least one SBA in the facility trained to manage neonatal resuscitation	1. Yes	2. No

Table 2: Checklist used in measuring process quality delivery service in Northern Ethiopia

Process quality variables: assessed using observation of mother during labour	Observed	
**A.** During Admission:		
Provider greets the woman and her companion (if present) in a cordial manner	1. Yes	2. No
Provider responds to mother immediate needs (thirst, hunger, cold/hot, need to urinate,	1. Yes	2. No
Provider ask mothers name	1. Yes	2. No
Provider ask her age	1. Yes	2. No
Provider ask about number of previous pregnancies/births	1. Yes	2. No
Provider ask any complications during labour and postpartum period	1. Yes	2. No
Provider ask other general medical problems	1. Yes	2. No
Provider ask use of medications	1. Yes	2. No
Provider ask about her HIV status	1. Yes	2. No
Provider estimate gestational age using last menstrual period	1. Yes	2. No
Provider ask mothers how frequently labour are occurring	1. Yes	2. No
Provider ask if her membranes ruptured: when, what color and what smell it had	1. Yes	2. No
Provider ask mothers whether she feels the baby’s movements	1. Yes	2. No
Provider ask mothers when the painful regular contractions began	1. Yes	2. No
Provider ask mothers whether she has experienced vaginal bleeding, fever, severe headaches, blurred vision, convulsion, or if any other problems the client is concerned	1. Yes	2. No
Provider records the information on clinical history chart	1. Yes	2. No
Ensures privacy with screen or curtain to separate the women from others at least during examination	1. Yes	2. No
Washes hands with running water and soap for 10-15 seconds and dries with an individual clean towel or allows hands to air dry	1. Yes	2. No
Explain each step	1. Yes	2. No
At least one encourage the woman to ask questions	1. Yes	2. No
Respond to the woman/ companion questions politely and in respectful manner	1. Yes	2. No
**B.** First stage of labour:		
Providers takes mother temperature	1. Yes	2. No
Provider takes mother pulse during admission/first stage labour	1. Yes	2. No
Provider measures mother blood pressure	1. Yes	2. No
Provider determines mother respiratory rate	1. Yes	2. No
Provider measures mother fundal height	1. Yes	2. No
Provider determines foetal lie and presentation	1. Yes	2. No
Provider identifies degree of decent by abdominal palpation (from five to zero fingers above the pubis)	1. Yes	2. No
Provider evaluates uterine contractions (frequency and duration over a 10-minute period)	1. Yes	2. No
Provider Auscultates foetal heart rate (FHR)	1. Yes	2. No
During physical examination, providers:		
Puts sterile gloves on both hands	1. Yes	2. No
Carefully inserts two fingers of the exam hand	1. Yes	2. No
Assess cervical examination	1. Yes	2. No
Assesses molding	1. Yes	2. No
Assesses station of presenting part	1. Yes	2. No
Assesses position	1. Yes	2. No
Gloves are removed after being immersed in 0.5% chlorine solution and placed in a leak-proof container	1. Yes	2. No
Records all information on the clinical records	1. Yes	2. No

Provider start partograph to follow progress of labour	1. Yes	2. No
Infection prevention practice of providers:		
Cleanse the vulva with antiseptic solution before performing vaginal examination	1. Yes	2. No
Performs limited vaginal examination (e.g, every four hours or as indicated)	1. Yes	2. No
Uses sterile gloves when performing vaginal examination or when in contact with body fluids	1. Yes	2. No
Rupture of membranes is not performed routinely	1. Yes	2. No
Provider prepares to assist the birth:		
Has the delivery pack and other essential material and ready to assist the delivery (delivery set, episiotomy set, newborn resuscitation set, glove, delivery gown, apron, mask)	1. Yes	2. No
Prior to delivery, washes hands with running water and soap for 10-15 seconds and dries an individual clean towel or allows hands to air dry	1. Yes	2. No
Puts sterile double gloves on hands	1. Yes	2. No
Provider:		
At least once encourage woman to walk around	1. Yes	2. No
At least once encourage the woman to change position according to the desire and comfort	1. Yes	2. No
At least once encourage the woman to take light food or drink fluid in labour	1. Yes	2. No
Allow the woman to have her preference companion at labour room	1. Yes	2. No
Provide emotional support to woman during labour and delivery	1. Yes	2. No
At least once encourages her to empty her bladder	1. Yes	2. No
**C.** Second stage of labour: provider		
Holds the baby by the trunk and places the baby on a clean dry towel on the mother's abdomen	1. Yes	2. No
Dries baby vigorously and changes wet towel for a clean dry one	1. Yes	2. No
Ties or clamps cord when pulsations stop, or by 2-3 minutes after birth (no immediately after birth)	1. Yes	2. No
Cuts/clamps the umbilical cord using sterile scissors under gauze to prevent blood spurting	1. Yes	2. No
If the baby is breathing normally, passes the baby to mother for skin-to-skin contact on breast	1. Yes	2. No
Note the time of birth and records on partograph or in other records	1. Yes	2. No
**D.** Third stage of labour: provider,		
Palpate the mother's abdomen to rule-out the presence of a second baby	1. Yes	2. No
Tells the woman that she will receive an injection and administers 10 IU of oxytocin IM or 600 mcg misprostol orally within 1 minute of delivery	1. Yes	2. No
Place the other hand on the women's symphysis pubis (over the sterile towel)	1. Yes	2. No
Maintain firm traction on the cord and waits for the uterus to contract	1. Yes	2. No
Upon contraction, applies firm and sustained downward traction on the cord with counter traction above the pubis to guard the uterus, until the placenta is expelled	1. Yes	2. No
If this maneuver does not provide immediate results, stops applying traction, holding the cord and clamp until the next contraction	1. Yes	2. No
Repeats control cord traction during contraction while simultaneously applying counter traction above pubis to guard uterus	1. Yes	2. No
With both hands, assists in the expulsion of the placenta by turning it over in the hands without applying traction twisting the membranes	1. Yes	2. No
Massage the uterus with one hand one sterile cloth over the abdomen until it contracts firmly	1. Yes	2. No
Checks to see whether the placenta is complete (maternal and foetal sides, plus membranes, lobs)	1. Yes	2. No
**E.** Immediate postpartum periods: provider,		
Make sure that the woman is comfortable (clean, hydrated and warmly covered)	1. Yes	2. No

Ensures that the baby is well covered	1. Yes	2. No
Administer vitamin K to newborn	1. Yes	2. No
Provides tetracycline eye ointment 1% prophylaxis to newborn	1. Yes	2. No
Observe breast feeding initiated within the first hour after birth	1. Yes	2. No
Weight the baby	1. Yes	2. No
Mother and newborn kept in same room after delivery	1. Yes	2. No
Discards the placenta in a leak-proof container with a plastic liner	1. Yes	2. No
Disposes of medical waste (gauze, etc) in a plastic container with a plastic liner	1. Yes	2. No
Puts the soiled linen in a leak-proof container	1. Yes	2. No
Places all reusable instruments in a 0.5% chlorine solution for 10 minutes	1. Yes	2. No
Disposes needle and siring in a puncture-resistance container, without removing, recapping or breaking the needle	1. Yes	2. No
Gloves are removed after being immersed in 0.5% chlorine solution and placed in a leak-proof container	1. Yes	2. No
Washes hands with running water and soap for 10-15seconds and dries with an individual clean towel or allows hands to air dry after conducting delivery	1. Yes	2. No
Provider monitors the mother and newborn after birth:		
Baby's breathing condition and breastfeeding during immediate postpartum period	1. Yes	2. No
Vaginal bleeding assessed during immediate postpartum period before discharge	1. Yes	2. No
Bladder distension assessed during immediate postpartum period before discharge	1. Yes	2. No
Blood pressure measured during immediate postpartum period before discharge	1. Yes	2. No
Uterine contraction assessed during immediate postpartum period before discharge	1. Yes	2. No
Pulse of mother measured during immediate postpartum period before discharge	1. Yes	2. No
Health condition of the mother assessed during immediate postpartum period before discharge	1. Yes	2. No
Assists the woman with breastfeeding after birth	1. Yes	2. No
Asks the woman if she has urinated and encourages her to do so whenever she wishes after birth	1. Yes	2. No
Records the information on the women's clinical record and reports any abnormalities after birth	1. Yes	2. No

Table 3: Interview guides used in measuring output quality (level of Satisfaction and EmONC utilization measures) in Northern Ethiopia study, 2015

Output quality variables		
**no**	**A. Satisfaction of mother (n=216): using exit interview (total 26 items)**	**Response**
1	The distance from your home to the nearest health facility is:	1. Long<br>2. Quite long<br>3. Short
2	To get transport from your home to this health facility is:	1. Difficult<br>2. Moderately difficult<br>3. Not at all difficult
3	The cost of transportation from your home to this health facility is:	1. Not at all affordable<br>2. Moderately affordable<br>3. Affordable
4	The fees you are charged at this health facility is:	1. Not at all affordable<br>2. Moderately affordable<br>3. Affordable
5	The opening hours at of this health facility is:	1. Not at all Suitable<br>2. Moderately suitable<br>3. Suitable
6	The doctors and midwives at this health facility are:	1. Not at all available<br>2. Moderately available<br>3. Available
7	The people who work in this health facility are:	1. Not very honest<br>2. Fairly honest<br>3. Honest
8	The doctors and midwives are Capable;	1. Not capable of finding out the problem with a pregnancy<br>2. Fairly capable<br>3. Incapable
9	How you obtain drugs:	1. With difficulty<br>2. With relative ease<br>3. Easily
10	The effectiveness of the medicine supplied by this facility is:	1. Not good<br>2. Fair<br>3. Good
11	The equipment is Adequate;	1. Inadequate for detecting diseases related to pregnancy<br>2. More or less adequate
12	The waiting rooms, examination rooms and delivery rooms are:	1. Inadequate<br>2. More or less adequate<br>3. Adequate
13	The pregnant women cared for:	1. Do not recover well<br>2. Recover relatively well<br>3. Recover well
14	The doctors and midwives examine you:	1. Not very Well<br>2. Moderately well<br>3. Well
15	The doctors and midwives are	1. Not very open with the pregnant women<br>2. Fairly open<br>3. Open
16	The doctors and midwives are	1. Not very compassionate towards pregnant women<br>2. Fairly compassionate<br>3. Compassionate
17	The doctors and midwives are	1. Not at all respectful towards pregnant women

		2. Fairly respectful<br>3. Respectful
18	The time that the doctors and midwives devote you is:	1. Inadequate<br>2. More or less adequate<br>3. Adequate
19	How do rate the privacy given to you during child birth	1. Inadequate privacy during examination by the midwife/ nurse<br>2. More or less adequate privacy<br>3. Adequate privacy
20	The number of doctors and midwives is:	1. Inadequate<br>2. More or less adequate<br>3. Adequate
21	The doctors and midwives are	1. Not well suited to deliver pregnant women<br>2. Relatively well suited<br>3. Well suited
22	Previous expectation toward quality care given at this facility	1. Poor<br>2. Moderate<br>3. Good
23	Currently received care	1. Poor service more than expected<br>2. Moderate<br>3. Good service more than expected
24	Are you satisfied with waiting time in this facility:	1. Un satisfied<br>2. Fairly satisfied<br>3. Very satisfied
25	Cleanliness of facility	1. Inadequate<br>2. More or less adequate<br>3. Adequate
26	The doctors and midwives involve you in decision making:	1. Not involved<br>2. Moderately involved<br>3. Totally involved

Table 4: Checklist used in assessing emergency obstetric and newborn care utilization in the past three months prior to study periods which is filled from maternity registers.

**Facility Case Summary Form for EmONC practice (Data extraction)**			
	**B. Emergency Obstetric utilization past 3 months prior to study periods: using record review Yes/no questions using record review at health facility (n=32); total 9 items**		
1	Parenteral antibiotics administered	1. Yes	2. No
2	Parenteral oxytocin administered	1. Yes	2. No
3	Parenteral anticonvulsants administered	1. Yes	2. No
4	Manual removal of placenta done	1. Yes	2. No
5	Removal of retained products (e.g., manual vacuum aspiration) done	1. Yes	2. No
6	Assisted vaginal delivery (e.g., vacuum extraction, breech delivery) done	1. Yes	2. No
7	Newborn resuscitation done	1. Yes	2. No
8	Blood transfusion done	1. Yes	2. No
9	Caesarian delivery (operative delivery) conducted	1. Yes	2. No
